# Using rare earth elements to constrain particulate organic carbon flux in the East China Sea

**DOI:** 10.1038/srep33880

**Published:** 2016-09-27

**Authors:** Chin-Chang Hung, Ya-Feng Chen, Shih-Chieh Hsu, Kui Wang, Jian Feng Chen, David J. Burdige

**Affiliations:** 1 Department of Oceanography, and Asia-Pacific Ocean Research Center, National Sun Yat-sen University, Kaohsiung, 80424 Taiwan; 2 Department of Ocean, Earth and Atmospheric Sciences, Old Dominion University, Norfolk, VA, 23529 USA; 3 Research Center for Environmental Changes, Academia Sinica, Taipei 115, Taiwan; 4 Laboratory of Marine Ecosystem and Biogeochemistry, State Oceanic Administration, Hangzhou, 310012, PR China

## Abstract

Fluxes of particulate organic carbon (POC) in the East China Sea (ECS) have been reported to decrease from the inner continental shelf towards the outer continental shelf. Recent research has shown that POC fluxes in the ECS may be overestimated due to active sediment resuspension. To better characterize the effect of sediment resuspension on particle fluxes in the ECS, rare earth elements (REEs) and organic carbon (OC) were used in separate two-member mixing models to evaluate trap-collected POC fluxes. The ratio of resuspended particles from sediments to total trap-collected particles in the ECS ranged from 82–94% using the OC mixing model, and 30–80% using the REEs mixing model, respectively. These results suggest that REEs may be better proxies for sediment resuspension than OC in high turbidity marginal seas because REEs do not appear to undergo degradation during particle sinking as compared to organic carbon. Our results suggest that REEs can be used as tracers to provide quantitative estimates of POC fluxes in marginal seas.

Continental margin seas occupy only a small portion (~8%) of the surface area of the ocean^1^, but they contribute about 30% of global primary production (PP)^2^. It has been suggested that marginal seas are an important organic carbon source to the open ocean because of higher nutrient inputs and PP as well as higher particulate organic carbon (POC) stocks as compared to those in the open ocean^3^. Generally, marginal seas are thought to profoundly affect marine carbon cycling and fisheries^4^,^5^,^6^.

The East China Sea (ECS) (Fig. 1) has been regarded as a sink (10–30 Mt C yr^−1^, where 1 Mt = 10^12^ g) for atmospheric carbon dioxide based on observations of CO_2_ air–sea exchange^7^,^8^,^9^,^10^,^11^. Model-estimated organic carbon (OC) burial on the broad ECS shelf (0.4 × 10^6^ km^2^) is 7–10 Mt C yr^−1^. The net imbalance of OC fluxes in the ECS amounts to approximately 10–20 Mt C yr^−1^ based on the difference between this input and burial. The estimated amount of OC transported offshore, which ranges from 2 to 12 Mt C yr^−1^ 4,12, seems to be insufficient to account for the deficit, which raises the question as to the fate of the extra carbon. Sinking particle fluxes of POC have been calculated in the inner and middle shelves of the ECS^13^,^14^,^15^, but direct observations of POC fluxes are limited^1^,^13^,^15^.

Recently, Hung *et al*.^1^ measured PP and the sinking particle POC flux in the ECS and found that some measured POC fluxes (720–7300 mgC m^−2^ d^−1^) were indeed higher than PP values (340–3380 mgC m^−2^ d^−1^). This suggested that resuspended sediments may also contribute to the measured POC flux, and that this contribution must be appropriately constrained. Hung *et al*.^1^ used a vertical mixing model to correct for the effects of active resuspension, and reported that approximately 27 to 93% of the measured POC flux in the ECS might be due to resuspension of bottom sediments. The large range of resuspension rates may be partly due to assumptions in the mixing model regarding POC composition, and because Hung *et al*.^1^ ignored organic matter degradation as particles sink in water column. Furthermore, it is expected that fine sediment particles are likely to undergo greater resuspension than large particles, and therefore, the mixing model should not simply consider bulk sediment. To further examine this problem here we chose to use REEs to estimate the contribution of resuspension to the sinking POC flux in the ECS. This approach was taken in part because REEs have been used in a number of different environmental settings^16^,^17^ as proxies for different sediment sources.

## Results

### Hydrographic and biogeochemical data

Vertical distributions of temperature, salinity, nitrate, chlorophyll-a (Chl *a*), POC and total suspended matter (TSM) concentrations in the inner (e.g., stations E1 and E5) and outer shelf of the ECS (e.g., stations E14 and E34) are shown in Fig. 2. The hydrographic settings are similar to previous studies with low salinity, high nutrient, high surface Chl *a* and high TSM concentrations occurring on the inner shelf, and high salinity, low nutrient and low surface Chl *a*, POC and TSM on the outer shelf (stations E14 and E34)^1^,^5^,^11^. In summer the water column at stations E5, E14 and E34 was stratified, with sub-surface maximum concentrations of Chl *a* and POC within the depth of the euphotic zone (E5:35 m, E14:72 m, E34:70 m, Fig. 2). A pronounced feature in the inner shelf is that TSM concentrations increased with increasing depth, suggesting the possible occurrence of sediment resuspension in the bottom waters.

### Uncorrected POC flux and PP in the ECS

A high POC flux (4846 mg C m^−2^ d^−1^), uncorrected for sediment resuspension, was observed in the inner shelf (E5) and low uncorrected POC fluxes (262–356 mg C m^−2^d^−1^) were observed in the outer shelf (E14 and E34) (Table 1). This is analogous to previous investigations where a high POC flux in the inner shelf gradually decreased towards the outer shelf^1^,^13^. PP was also high (1682 mg C m^−2^d^−1^) within the inner shelf and lower (i.e., 748 and 1480 mg C m^−2^d^−1^) on the outer shelf (Table 1). In comparison, Hung *et al*.^1^ observed a high POC flux in the inner shelf that could have been caused by high fluvial POC discharge from the Changjiang River. Suspended particle discharge from the Changjiang River may largely affect the POC flux calculations for the inner shelf if the study area has low salinity water overlying the surface layer. Due to the possible effects of horizontal particle transport from the Changjiang River during the time of our cruise, we chose not to deploy a sediment trap at station E1. The POC flux at station E5 is substantially higher than the PP value and the *e* ratio (POC flux/PP, Table 1) at stations E14 and E34 in the ECS are higher than values observed at similar oceanographic settings^18^. These observations along with salinity distribution data (high salinity of the surface waters; Fig. 2) suggest that the effect of fluvial particle discharge is not likely important on the outer shelf. We therefore suspect that sediment resuspension may contribute to the high measured POC flux and, hence, the POC fluxes need to be re-evaluated in light of active sediment resuspension^1^. Below, we use a vertical mixing model to correct our measured POC fluxes for sediment resuspension.

### A vertical particle-mixing model to correct POC flux

A two end-member mixing model was used to evaluate the resuspension of bottom sediments^19^,^20^,^21^. The end-members of the model are as follows: (1) surface particles that are characterized by high POC content (%) and low TSM; and (2) sediments consisting of low POC content (%) and high TSM. The mixing model was evaluated using:


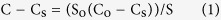


where C is the observed POC content (%) in the suspended particles, S_o_ is the total weight of surface phytoplankton (mg L^−1^), S is the total weight of observed suspended particles (mg L^−1^) in the surface water, C_o_ is the POC content of surface phytoplankton (unknown), and C_s_ is the POC content we measured in surface sediment (0–2 cm). Using the measured C_s_ values and assuming a reasonable surface phytoplankton weight (i.e., S_o_ = 0.5 mg L^−1^), C_o_ values (Table 2) were estimated by plotting (C-C_s_) Versus the reciprocal of the observed TSM (1/S) and forcing the best-fit line through the origin (Fig. 3). The predicted C_o_ values (phytoplankton POC content) ranged from 7.0 to 28.1%, which are similar to the POC contents (8.1–16.8%) for the predominate phytoplankton species in the ECS^1^.

Next, the two end-member values (C_s_ and C_o_) are used to estimate the ratio of resuspended particles to total sinking particles in the expression:


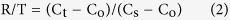


where R/T is the ratio of resuspended particles to total sinking particles collected by a sediment trap, and C_t_ is the organic carbon content (2.3, 1.4, and 1.5% at E5, E14 and E34, respectively) of sinking particles. Finally, the POC flux corrected for sediment resuspension is then calculated as:


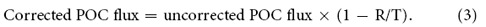


The results of these computations for the R/T ratios, and the uncorrected and corrected POC fluxes are summarized in Table 2. The predicted R/T ratio in trapped particles of the ECS ranged from approximately 82% to 94% with higher values in the inner shelf and lower values in the outer shelf, suggesting that sediment resuspension is a ubiquitous phenomenon in the ECS. The calculated resuspension ratios (82–94%) are similar to previously reported values in the inner and middle shelves of the Yellow Sea (70–90%)^15^ and in the ECS (57–93%)^1^.

The corrected POC fluxes (48–292 mgC m^−2^ d^−1^) are lower than the PP values (Table 3) in the ECS, indicating that the original uncorrected POC fluxes were indeed elevated owing to sediment resuspension. It is not clear, however, whether the vertical mixing model using POC as a proxy for resuspension is a robust tool^1^ because it does not consider sinking organic matter degradation and the importance of particle size on sediment resuspension. In the next section, we employ the rare earth elements (REE) to re-examine these issues.

### Rare Earth Elements as a proxy

The distribution of shale-normalized REE concentrations in suspended, sinking particles, and surface sediments in the ECS are shown in the Fig. 4. Most of normalized REEs show low values in surface water suspended particles and increase with increasing water depth, reaching maximum observed values in sediments. Exceptions include Ce at stations E5 and E34, and La at station E5.

The REE content of size fractioned sediments (<20 μm, 20–50 μm, 50–330 μm, and >330 μm) near station E1 is shown in the Fig. 4D. The <20 μm, 20–50 μm, and >330 μm fractions had, on average, the highest amounts of REEs, whereas the 50–330 μm fraction contained the lowest amount of REEs. The fraction of REEs in the >330 μm size fraction ranged from 25 to 31% of the total REE content in the sediments. Researchers have reported that the size distribution of sinking particles can range from 1 μm to hundreds of μm and sinking particles in the <330 μm size fraction (<20 μm + 20–50 μm + 50–330 μm) are still abundant^22^. Because particles in this size fraction are likely to make up the bulk of the resuspended bottom sediments over any appreciable depths in the water column, we used a similar approach to the two-end member POC model described above where the concentration of REEs in the <330 μm size fraction was employed to calculate the R/T ratio for sinking particles. The R/T ratios based on individual REEs (Pr, Nd, Sm, Eu, Gd, Tb, Dy, Ho, Er, Tm, Yb, and Lu) ranged from 0.66 to 0.88 at E5, from 0.37 to 0.67 at station E14, and from 0.14 to 0.58 at station E34, respectively (Table 4). The average resuspension ratios at E5, E14, and E34 based on all REEs are 79 ± 9%, 54 ± 10%, and 30 ± 15%, respectively. The resuspension ratios based on REEs are lower than those obtained using the POC method (i.e., 2% at E5, 56% at E14, and 86% at E34, respectively). Consequently, the average POC fluxes, corrected using the REEs resuspension ratios, at E5, E14, and E34 are 998 ± 319, 120 ± 27, and 250 ± 52 mg C m^−2^ d^−1^, respectively (Table 4).

## Discussion

### OC content in suspended particles, sinking particles and sediments

Some researchers have reported a loss of bulk organic carbon into the dissolved fraction in sediment traps that ranged from 0.8% to 2% per hour^23^. If the maximum degradation rate (2% per hour) is applied to sinking surface phytoplankton collected in sediment traps, the estimated resuspended fractions in trapped particles on the inner shelf (e.g. E5) are 92% and 90% (versus 94%) if 20% or 40% of bulk biogenic particles are degraded during sinking and/or repeated active resuspension.

Besides the degradation of biogenic particles during sample collection, phytoplankton biomass in the surface waters may change before sampling and the estimated value of the R/T ratio may therefore be affected. If we decrease S_o_ from 0.50 to 0.3 mg L^−1^, (Table 2) at a fixed value of C_t_ the R/T ratio at station E5, for example, increases from 94 to 96%. If we increase S_o_ from 0.5 to 0.7 mg L^−1^ (Table 2), again at fixed value of C_t_, the R/T ratio decreases from 94 to 92%. An analogous approach can be used to examine the impact of sediment OC degradation on the R/T ratio; if 20% or 40% of the resuspended sediment at E5 are degraded, the estimated R/T ratio in the trapped particles will change from 94% to either 93.5% or 93.1% respectively, suggesting that uncertainty in the OC content of surface biogenic particles has a greater impact on the calculated R/T ratio than does that associated with the sediment OC concentration.

### Rare earth elements as a proxy to calibrate POC flux

As addressed above, the two end-member mixing model using organic carbon does not explicitly consider particles degradation in the sediment traps or in the water column as the particles sink. Uncertainty in the calculation also stems from uncertainties in estimates of the carbon content of the surface biogenic biomass that contributes to the trap material. Here, time series data for surface biogenic carbon concentrations along with sediment trap results would be necessary to address these questions.

In contrast, REEs as proxies for sediment resuspension are less likely to be impacted by the degradation processes discussed above, and consequently, are expected to produce more consistent R/T ratios than those obtained with the organic carbon approach. For example, the estimated resuspended fraction in sediment trap particles in the inner shelf (station E5) using eleven REEs was 79 ± 9%, i.e., with less than 10% standard deviation (the R/T ratio for Tm was excluded here because this physically impossible ratio may be due to analytical uncertainty because this HREE is monoisotopic and occurs at very low abundances.). The same was the case at E14, with R/T = 54 ± 10% for all 14 naturally occurring REEs. A large relative variation in the R/T ratio was observed at E34 with 30 ± 15% (about 50% standard deviation). This average R/T ratio was computed by excluding from this calculation individual REEs that exhibited exceptionally high and low (Gd and Lu), or negative (i.e., Nd), R/T ratios. These specific R/T ratios (e.g., Tm at station E5, and Nd, Gd, Tb, Ho, and Lu at station E34) are difficult to interpret and may reflect small-scale variability in the composition of suspended particles that contribute to the sinking particle flux.

For example, surface coatings of organic matter and oxides of Mn and Fe, may be responsible for the removal and fractionation of some REEs between suspended particles and seawater^24^,^25^,^26^. Specifically the preferential removal of tri-valent LREEs over trivalent HREEs may occur because of uptake of Ce and LREEs on particle surfaces as a result of *in situ* oxidation of dissolved Ce(III) to particulate Ce(IV)^26^. This type of fractionation of tri-valent REEs is consistent with particle/solution models^26^.

By combining Ce concentration data with information about the Ce (III) oxidation rate a residence time of about thirteen days was estimated for suspended particles in the Sargasso Sea^26^,^27^. In contrast, the residence time of sinking particles in the ECS is less than two days (assuming a particle sinking rate of 100 m/day and a water depth of 100 m, the residence time is about 1 day). Since this time is much less than the reaction time (~13 days) for REE uptake from seawater onto particles, the affinity of different REEs to organic matter in sinking particles probably does not significantly impact our calculations.

Alternatively, there may be a third source of REEs (i.e., terrestrial or riverine input) that is not accounted for in the two end-member mixing model, although the high salinity of the waters at these stations (Fig. 2) argues against such an explanation. Whereas more work is needed to better understand the reasons for these apparent outliers in our results, overall our results suggest that REEs, in general, are likely useful tracers of the impact of sediment resuspension on sinking particle fluxes in both high turbidity inner shelf waters and in outer shelf waters.

To reconcile the differences in the R/T ratios determined with the OC model versus the REEs model, we use the REEs R/T ratio computed with Eq (2) and measured carbon content of the sediments to first recalculate C_o_. With this approach C_o_ (i.e., the POC concentration of surface phytoplankton) at station E5 decreases to 8.4% for the REE R/T ratio of 0.79. Similarly, C_o_ at stations E14 and E34 also decrease to 2.9% and 2.1%, using REE R/T ratios of 0.54 and 0.30, respectively. These results suggest that either POC in surface biogenic particles is easily degraded in summer months during sinking or that POC is effectively recycled in the ECS water column.

At the same time, such decreases in C_o_ at each station also require that the S_o_ values increase, because the slope of the regression lines in Fig. 3 (see Eq. 1) is given by S_0_(C_0_-C_S_). The re-calculated S_o_ values (1.74, 1.25, and 2.58 mg L^−1^ at stations E5, E14, and E34, respectively) are higher than the values used in Table 2 (i.e., 0.3 to 0.7 mg L^−1^) but are not unreasonable values for the ECS^14^. These observations further suggest the need for better synoptic measurements of suspended matter concentrations (both total and carbon content) and sediment trap fluxes.

Several factors make REEs suitable tracers for correcting sediment trap fluxes for sediment resuspension. First, REEs are not significantly involved in bio-uptake process as compared to other trace metals (e.g., Fe, Zn, Cd) that are taken up by phytoplankton^28^. Secondly, REEs are subject to far less degradation during active resuspension as compared to organic matter derived from either sediments or surface water productivity. One can see a remarkable decrease in the REE content and shale-normalized REEs patterns when sediments are compared to suspended matter from bottom waters, and when suspended matter from bottom waters are compared to suspended matter from surface waters, which strongly suggests active vertical particle mixing^26^,^29^−^31^. Third, REEs among suspended particles, sinking particles, and sediments exhibit distinct differences in their shale-normalized REE patterns, which allow them to be used as suitable tools for differentiating between material derived from sinking surface particles and that derived from resuspended bottom sediments (Fig. 4).

The REEs-corrected POC fluxes at E5, E14, and E34 are 998 ± 319, 120 ± 27, and 250 ± 352 (mgC m^−2^ d^−1^), respectively (Table 4). The corresponding *e*-ratios computed for stations E5, E14, and E34 are 0.59 ± 0.19, 0.16 ± 0.04, and 0.17 ± 0.04, respectively. For comparison, Hung *et al*.^32^ used nitrate reductase measurements to estimate nitrate uptake, another tracer for new production, in the ECS. The nitrate uptake rate/PP ratios they determined (0.15 to 0.5), which should be roughly equal to the *e* ratio, are quite similar to the *e* ratios estimated here with REE-corrected POC fluxes. This suggests that REEs-corrected POC fluxes in the ECS may be more reasonable than POC-corrected POC fluxes.

## Materials and Methods

**Figure 1:**
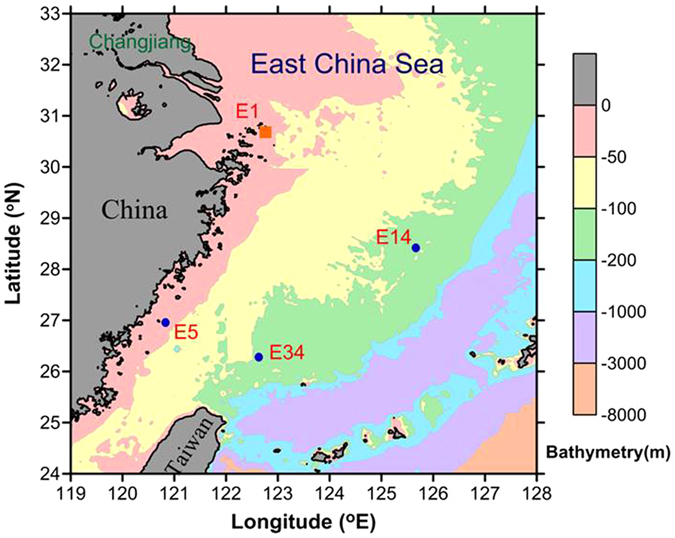
Sampling locations in the East China Sea. Blue dots represent the surface sediment stations and the sediment trap deployment station in summer in 2013. Station E1 was in November 2013. (The map was created using Surfer software v.12 Surfer (Golden Software) http://www.goldensoftware.com/home/terms-of-use).

**Figure 2:**
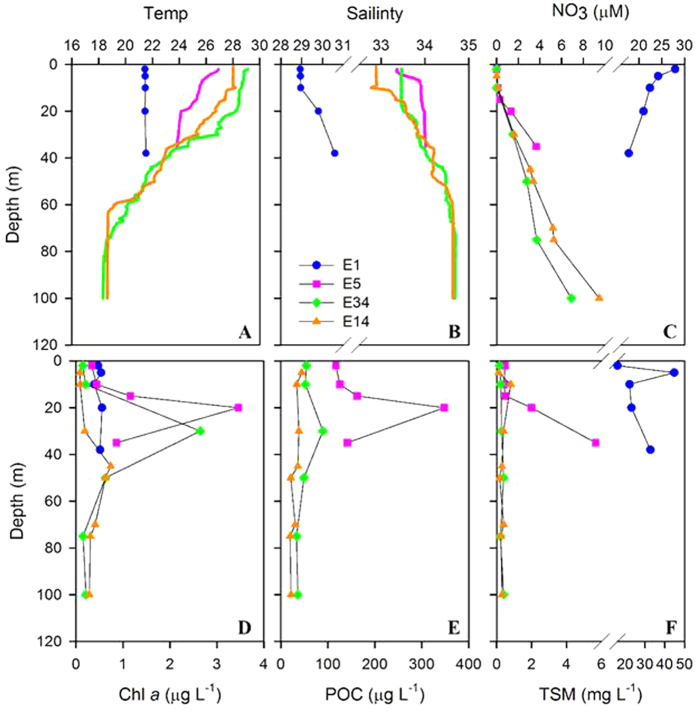
Depth profiles for temperature (Temp), salinity, nitrate (NO_3_), chlorophyll (Chl *a*), particulate organic carbon (POC) and total suspended matter (TSM) concentrations in the inner shelf (E1, E5) and the outer shelf (E34 and E14) of the East China Sea. Note: POC data are not available at station E1.

**Figure 3:**
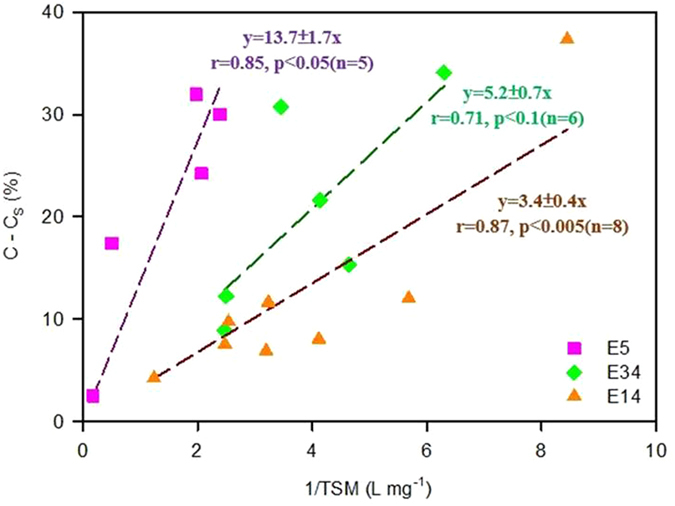
Relationships between C-C_s_ and 1/TSM in the ECS. The regression lines are forced through the origin in order to use a POC value in sediments.

**Figure 4:**
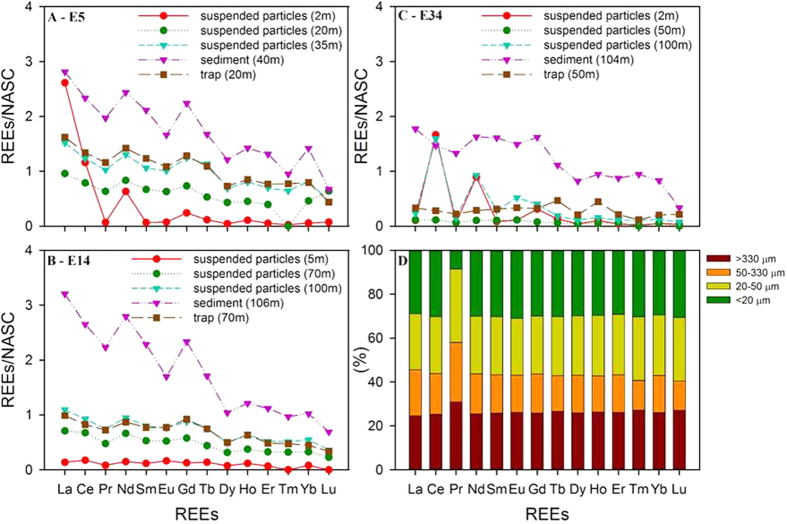
(**A**) Distribution of shale-normalized REEs in suspended particles at different depths, sinking particles, and sediments at stations E5, E14 (**B**), and E34 (**C**), respectively. (**D**) Distribution of REEs in size fractioned sediments near station E1. NASC represents REEs in the North American shale composite^31^.

Seawater samples were collected at stations E5, E14, and E34 aboard the R/V *OR-I* in the ECS in July 2012 and at station E1 in November 2013 (Fig. 1). Temperature and salinity were recorded using a SeaBird model SBE9/11 plus conductivity–temperature–depth (CTD) recorder. Distinct seawater samples were collected using Niskin bottles from different depths for measurements of chlorophyll *a* (Chl *a*) and POC concentrations^1^. Surface sediment samples (0–2 cm) near station E1 were collected using a box-core sampler and were immediately wet-sieved through sequential Nitex screens into three size fractions with cut-offs of 150, 50 and 20 μm. Size fractionated (>150, 50–150, 20–50, and <20 μm) sediments were subsequently rinsed with Milli-Q water and then re-filtered through polycarbonate filters prior to rare earth element (REEs) analysis as described below.

Seawater samples were filtered through pre-weighed GF/F filters for POC measurement and polycarbonate filters for measuring TSM and concentrations of REEs. Sinking particles were collected at 20 m (station E5), 70 m (E14) and 50 m (E34) by a floating sediment trap array. Detailed procedures have been reported in Hung *et al*.^1^ Concentrations of rare earth elements in the sinking particles, processed by the total digestion method using a mixture of the acids HF, HNO_3_, and HClO_4_, were determined by quadrupole-based inductively coupled plasma mass spectrometer given by Hsu and Lin^33^. The REE concentration data are presented by normalization to North American Shale Composite, NASC^34^. Mean Chl *a* data from water column samples was used to estimate primary production (PP) using the Vertically Generalized Production model of Behrenfeld and Falkowski^35^, since satellite derived chlorophyll values in marginal seas may not be reliable.

## Additional Information

**How to cite this article**: Hung, C.-C. *et al*. Using rare earth elements to constrain particulate organic carbon flux in the East China Sea. *Sci. Rep*. **6**, 33880; doi: 10.1038/srep33880 (2016).

## Figures and Tables

**Table 1:** Parameters of water depth, trap deployment depth, uncorrected (Un.) POC flux and primary production (PP) in the East China Sea.

Station	Water Depth (m)	Trap Depth (m)	Un. POC flux (mgC m^−2^ d^−1^)	PP (POC flux/PP)	e ratio
E5	40	20	4846	1682	2.88
E14	107	70	262	748	0.35
E34	104	50	356	1480	0.24

**Table 2:** Statistical data of linear regressions of POC values versus the reciprocal of total suspended matter concentrations in the ECS.

Station	Slope S_0_(C_0_-C_S_)	C_s_	C_0_ S_0_ = 0.5	C_0_ (Max) S_0_ = 0.3	C_0_ (Min) S_0_ = 0.7	R/T
E5	13.7	0.65	28.1	46.3	20.2	0.94
E14	3.4	0.15	7.0	11.4	5.0	0.82
E34	5.2	0.09	10.5	17.4	7.5	0.87

The unit of Cs and Co is %. Co (max) and Co (min) represent the maximum and minimum derived POC concentrations of phytoplankton.

**Table 3:** Detailed values of R/T, uncorrected POC flux and corrected POC flux in the ECS.

Station	POC flux^a^	POC flux^b^	POC flux^c^	e ratio^a^	e ratio^b^	e ratio^c^
		(mgC m^−2^ d^−1^)				
E5	4846	291.8	998 ± 319	2.88	0.17	0.59 ± 0.19
E14	262	48.2	120 ± 27	0.35	0.06	0.16 ± 0.04
E34	356	47.5	250 ± 52	0.24	0.03	0.17 ± 0.04

^a^Uncorrected POC flux.

^b^Corrected POC flux using OC method.

^c^Average corrected POC flux (average flux ± uncertainty) using REEs method.

**Table 4:** Estimated resuspension of trapped sinking particles and corrected POC flux in the East China using different rare earth elements.

Station REEs	E5 (R/T)	E14 (R/T)		E5	E14	E34
			E34 (R/T)	corr. POC flux (mgC m^−2^ d^−1^)		
Pr	0.77	0.40	0.17	1115	157	296
Nd	0.66	0.37	(−1.9)	1633	165	n.a.
Sm	0.84	0.45	0.22	790	145	277
Eu	0.87	0.55	0.22	649	118	276
Gd	0.73	0.50	(0.02)	1289	132	n.a.
Tb	0.87	0.54	0.49	635	121	182
Dy	0.80	0.61	0.29	955	103	254
Ho	0.79	0.67	0.58	1008	86	148
Er	0.78	0.55	0.28	1076	118	255
Tm*	(1.1)	0.66	0.14	n.a.	88	306
Yb	0.75	0.54	0.28	1226	120	258
Lu	0.88	0.67	(0.86)	601	86	n.a.
Average	0.79	0.54	0.30	998	120	250
Stdev	0.07	0.10	0.15	319	27	52

Values greater than 1 or less than zero are not physically realistic and may be due to other possible sources of REEs (other than the two used in our model) and/or analytical uncertainty in measured REEs concentrations. These were not used to estimate corrected POC fluxes (listed as n.a. in the three far right columns).
