# Corrigendum: Using rare earth elements to constrain particulate organic carbon flux in the East China Sea

**DOI:** 10.1038/srep37881

**Published:** 2016-12-09

**Authors:** Chin-Chang Hung, Ya-Feng Chen, Shih-Chieh Hsu, Kui Wang, Jianfang Chen, David J. Burdige

Scientific Reports 6: Article number: 3388010.1038/srep33880; published online: 09 27 2016; updated: 12 09 2016

The original version of this Article contained a typographical error in the spelling of Jianfang Chen, which was incorrectly given as Jian Feng Chen.

This has now been corrected in the HTML and PDF versions of the Article

